# Preparation and Characterization of PVA Alkaline Solid Polymer Electrolyte with Addition of Bamboo Charcoal

**DOI:** 10.3390/ma11050679

**Published:** 2018-04-26

**Authors:** Lidan Fan, Mengyue Wang, Zhen Zhang, Gang Qin, Xiaoyi Hu, Qiang Chen

**Affiliations:** 1 School of Civil Engineering, Henan Polytechnic University, Jiaozuo 454001, China; lidanfan@hpu.edu.cn; 2 School of Materials Science and Engineering, Henan Polytechnic University, Jiaozuo 454001, China; wangmengyue1111@sina.com (M.W.); zhangergou1014@sina.com (Z.Z.); yizhixinhai_hu@163.com (X.H.)

**Keywords:** alkaline solid polymer electrolyte, polyvinyl alcohol, bamboo charcoal, ionic conductivity, nickel-hydrogen battery

## Abstract

Natural bamboo charcoal (BC) powder has been developed as a novel filler in order to further improve performances of the polyvinyl alcohol (PVA)-based alkaline solid polymer electrolyte (ASPE) by solution casting method. X-ray diffraction patterns of composite polymer electrolyte with BC revealed the decrease in the degree of crystallinity with increasing content of BC. Scanning electron microscopy images showed pores on a micrometer scale (average diameter about 2 μm) distributed inside and on the surface of the membranes, indicating a three-dimension network formed in the polymer framework. The ionic conductivity was measured by the alternating-current (AC) impedance method, and the highest conductivity value of 6.63 × 10^−2^ S·cm^−1^ was obtained with 16 wt % of BC content and *m_KOH_*:*m_PVA_* = 2:1.5 at 30 °C. The contents of BC and KOH could significantly influence the conductivity. The temperature dependence of the bulk electrical conductivity displayed a combination of Arrhenius nature, and the activation energy for the ion in polymer electrolyte has been calculated. The electrochemical stability window of the electrolyte membrane was over 1.6 V. The thermogravimetric analysis curves showed that the degradation temperatures of PVA-BC-KOH ASPE membranes shifted toward higher with adding BC. A simple nickel-hydrogen battery containing PVA-BC-KOH electrolyte membrane was assembled with a maximum discharge capacity of 193 mAh·g^−1^.

## 1. Introduction 

Solid polymer electrolytes (SPEs) have been studied extensively in recent years for application in many electrochemical devices, such as rechargeable batteries [1], fuel cells [2], supercapacitors [3]. Polymer electrolyte membrane, also known as separator, plays an important role in the development of battery systems. The ionic conductivity (σ) and security are the greatest concerns for an electrolyte [4]. Compared to aqueous electrolyte (i.e., KOH), alkaline solid polymer electrolyte (ASPE), for use in nickel-hydrogen (Ni-MH) batteries, could provide solutions to the problems of high internal pressure upon charging [5], alkaline leakage [6], oxidation of negative electrode (hydrogen storage alloy) upon charge/discharge cycling [7] and high self-discharge rate [8]. Besides the ASPE features good processibility [9], and mechanical properties. To sum up, ASPE have been suggested to replace aqueous electrolytes [10].

Some studies have so far been focused on polyethylene oxide (PEO)-based electrolyte systems. Hassan et al. [11] reported the ionic conductivities of the PEO-KOH-H_2_O ASPEs reaching the level of 10^−5^ S·cm^−1^ with using in Ni-MH batteries. Chandrasekaran et al. [12] prepared an ASPE based on PEO + NaClO_3_ for using in Ni-MH batteries, and the ionic conductivity reached 10^−4^ S·cm^−1^. Although the PEO-based ASPEs could form a dimensionally stable membrane, but the conductivity was still too low for application in electrochemical devices [13]. Fortunately, polyvinyl alcohol (PVA) features great affinity to water, which could make desirable contribution to promoting ion conduction in aqueous-based SPEs [14]. Meanwhile, PVA becomes an attractive material for the use in the ASPEs due to its low cost, excellent film forming property and stability in an alkaline environment. This hydrophilic semi-crystalline polymer can become an effective electrolyte by doping with an alkaline (for example KOH) solution [15]. Lewandowski et al. [16] reported the ionic conductivities of a series of thin-film SPEs based on PVA-KOH-H_2_O reached the level of 10^−3^ S·cm^−1^ with using in Ni-MH batteries. Li et al. [17] prepared the ASPE based on tetraethylammonium hydroxide (TEAOH)-PVA for solid electrochemical capacitors, and achieved an ionic conductivity of 5 × 10^−3^ S·cm^−1^.

Yang et al. [18] reported a study based on the PVA-KOH-H_2_O system. The relation between the ionic conductivity and the chemical components of PVA-based electrolyte was investigated. The results indicated that the ionic conductivity of the PVA-KOH system strongly depended on the content of KOH and the water in the PVA matrix. If the water content in the composite electrolyte membrane increased, the more KOH would dissolve in the PVA matrix, which improved ionic conductivity directly [19]. However, the pure PVA-based ASPEs are easy to dehydrate, causing some drawbacks, for example, being crimped, distorted and brittle. Given the above, the applicability of pure PVA-based ASPEs becomes poor and electrochemical performances drop off [6].

In conclusion, modifying pure PVA-based ASPEs is necessary. At present, there are four frequently-used modification methods including copolymerization, blending, the utilization of plasticizer and the addition of inorganic fillers [20], with the fourth being the simplest operation method and lowest cost. It is observed that some achievements have been obtained by the addition of ceramic particle. Hema et al. [21] prepared and characterized an ASPE based on PVA-PVDF-SiO_2_ whose ionic conductivity achieved 9.4 × 10^−4^ S·cm^−1^. More et al. [22] reported PVA-Al_2_O_3_ electrolyte membrane, which had an ionic conductivity of 3.2 × 10^−4^ S·cm^−1^ as the weight ratio was 10:1 (PVA:Al_2_O_3_), and the mechanical strength was also improved. The addition of ceramic particles into the polymer matrix succeeded in reducing the crystallinity of the PVA, increased the amorphous phases of PVA matrix, then somewhat improved the ionic conductivities.

On the basis of current research, we developed a novel filler in order to further improve performances of the PVA-based ASPEs. We attempted to disperse the natural bamboo charcoal (BC) powder as filler in pure PVA, and reported the preparation and characteristic properties of the PVA-BC-KOH composite electrolyte. As a renewable environment-friendly material, BC has a wide application prospect because of hydrophilicity, simple fabrication technologies [23], low cost and excellent adsorption properties [24]. Otherwise, in this work, the Ni-MH battery was used to study the potential application of PVA-BC-KOH electrolyte membrane.

## 2. Experimental Section

### 2.1. Materials

PVA (model: 1799, AR) was purchased from Aladdin Reagent Company (Shanghai, China) with the molecular weight of about 75,000 g·mol^−1^. KOH (AR) was purchased from Tianjin Fuchen Chemical Reagent Factory (Tianjin, China). BC (fineness: 3 μm) was purchased from Zhejiang Wanglin Company (Jiangshan, China). All raw materials were used without further purification.

### 2.2. Preparation of the PVA-BC-KOH Electrolyte Membranes

The PVA-BC-KOH ASPEs were prepared by solution casting and the solvent evaporation technique. Stoichiometric amounts of PVA and BC were mixed together in a glass beaker and dissolved in 20 mL distilled water. The mixed solution described above was heated in a thermostat water bath at 85 °C for 30 min until solid components dissolved completely. It was followed by the addition of KOH solution at various concentrations, and the dosage of the raw materials are listed in the Table S1 (Supplementary Materials). After that, the solution continued to be stirred for 15 min. At this stage, PVA-BC solution was doped with KOH, forming the uniform solution eventually. Subsequently, the resulting solution was cast into a petri plate to obtain membrane of the desired thickness. The solvent was allowed to evaporate at ambient temperature till the formation of the thin-film. The thickness of the thin membrane was about 0.35 mm.

### 2.3. Morphology Investigation

The surface morphology of the PVA-BC-KOH composite membrane was investigated by a field emission-scanning electron microscope (FE-SEM, Merlin Compact. Carl Zeiss NTS GmbH, Jena, Thuringia, Germany). Prior to the observation, the sample membranes were freeze-dried and fractured in liquid nitrogen, then the specimens were treated by gold-sputtered in an ion sputtering instrument (SBC-12, KYKY Technology Co. Ltd., Beijing, China).

### 2.4. XRD Analysis

The X-ray diffraction (XRD) measurements were performed on the PVA-BC-KOH membranes to examine the crystal structure by an X-ray diffractometer (D-500, Siemens Ltd., Munich, Germany) at wavelength 0.154 nm. The XRD spectrum of every sample was recorded in the angles (2θ) of 10–70°. The square pieces (2 cm × 2 cm), which were sandwiched between two glass sheets with a partition in the middle of them to let the samples breath freely. Finally, the sample structure was tested after drying for 12 h.

### 2.5. Moisture Content Measurement

The swelling of membranes was estimated by moisture content ($W_{H_{2}O}$) from the mass change before and after complete dryness of the membrane. The ASPE membrane was weighed and then was dried overnight in a vacuum oven at 60 °C, the moisture content was calculated using Equation (1):

(1) $$W_{H_{2}O}\%=\frac{\left(W_{wet}-W_{dry}\right)}{W_{wet}}\times 100\%$$

where *W_wet_* and *W_dry_* are the wet and dry membrane weights, respectively.

### 2.6. Thermal Analysis

The thermo-gravimetric analysis (TGA) was performed with thermal analyzer (DSC-Q10, TA Co., New Castle, PA, USA) under argon atmosphere. The thermal behavior of every sample (6–10 mg) was investigated in the temperature range of 20–600 °C. The sample membranes were tweezered and washed quickly in deionized water three times to remove the KOH solution on the surface. And then they were dried in an oven at 60 °C for 6 h and cut into pieces with scissors. Moisture Content Measurement.

### 2.7. Measurement of Ionic Conductivity

The alternating-current (AC) impedance technique was used to measure the ionic conductivities of the PVA-BC-KOH ASPEs with the aid of an electrochemical workstation (Parstat2273, Princeton Applied Research Co., USA). Round pieces (radius = 0.65 cm), which were cut from the PVA-BC-KOH ASPEs, were sandwiched between two stainless steel (SS) blocking electrodes to form a cell. Every sample was placed at testing temperature for 30 min before measurement. The bulk resistance (Rb) of every sample was obtained with frequency ranging from 10 kHz to 100 mHz. The corresponding Rb was used to calculate the ionic conductivities. Also, the Rb for the sample membrane was recorded under different temperature to know the variation trend of ionic conductivities following temperature. Experimental temperatures were maintained within ± 0.1 °C by an oven. XRD Analysis.

### 2.8. Measurement of the Electrochemical Stability Window

Using the same SS/PVA-BC-KOH ASPE/SS cell of AC impedance and electrochemical workstation, the electrochemical stability window can be obtained by cyclic voltammetry (CV) for setting a cycle voltage region from −2 V to 2 V at a scan rate of 10 mV·s^−1^. Experimental temperature was maintained at 30 °C.

### 2.9. The Assembly of Ni-MH Battery

This study adopted hydrogen storage alloy based on cobalt as the negative electrode, and Ni(OH)_2_ was used for the positive electrode. The PVA-BC-KOH ASPEs were sandwiched between the negative electrode and the positive electrode to form the simulated polymer battery, and then the feasibility of the PVA-BC-KOH ASPEs in Ni-MH battery was tested. The Ni-MH battery was charged at 60 mA for 3 h, and then discharged at 9 mA until the voltage reduced to about 0.1 V by a battery test instrument (CT 3008, Neware Technology Co. Ltd., Shenzhen, China). The battery was observed for 20 cycles by galvanostatic method.

## 3. Results and Discussions

### 3.1. Morphology

**Figure 1:**
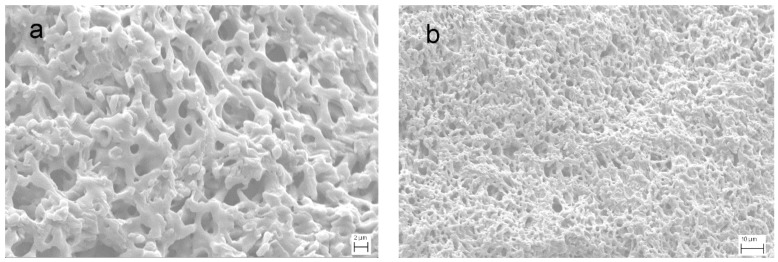
The FE-SEM micrographs of polyvinyl alcohol (PVA)-bamboo charcoal (BC)-KOH electrolyte membrane. (**a**) High magnification; (**b**) low magnification; *m_KOH_*:*m_PVA_* = 2:1.5 and BC content of 16 wt % at 30 °C.

The FE-SEM micrographs of PVA-BC-KOH ASPEs are shown in Figure 1a,b. The morphology of PVA-BC-KOH ASPE membranes are similar and the sample with BC content of 16 wt % was taken as a representative. The FE-SEM micrographs reveal the relatively uniform pores with diameter about 2 μm in PVA-BC-KOH ASPEs, and the polymer framework forms a three-dimension network. The above two structures allowed more KOH electrolyte to be retained with good water absorbing capacity, thus increased the number of the OH^−^ in the PVA matrix, which enhanced the ionic conductivity of PVA-BC-KOH ASPEs.

### 3.2. XRD Analysis

**Figure 2:**
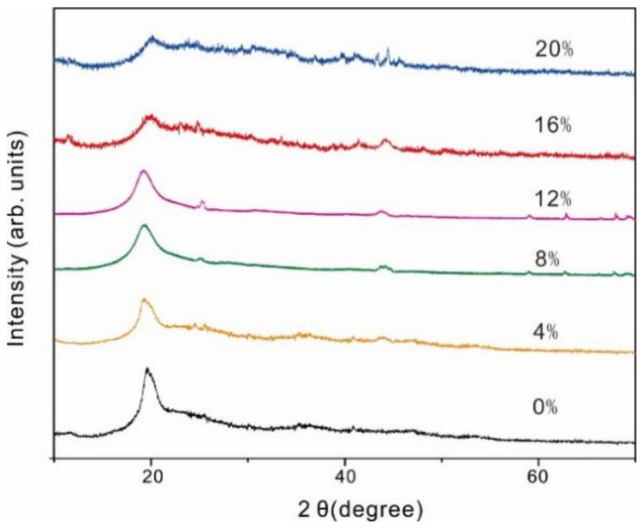
The X-ray diffraction (XRD) patterns of PVA-BC-KOH electrolyte membranes.

XRD has been used for confirming the crystallinity change of polymers, which is closely related to ionic conductivity for SPEs. The XRD spectra of PVA-BC-KOH membranes at ambient temperature with the content of BC of 0, 4, 8, 12, 16 and 20 wt % were examined. As shown in Figure 2, the pure PVA is known to exhibit a semi-crystalline structure with an intense peak at 2θ angle of 19.6°, corresponding to 101 crystallographic plane of PVA. However, it is clearly observed that the peak intensity of pure PVA greatly reduces with the increase of BC content, which may be attributed to the decrease of PVA crystallinity resulting from the separation of polymer chains followed by reorganization of its structure [22]. It can be concluded that the addition of BC into PVA polymer matrix enlarged the amorphous region [25]. The increase of the amorphous region was beneficial to local PVA chain segment motion and water uptake, which promoted the ions migration thus improving the ionic conductivities of PVA-BC-KOH membranes [26]. The peaks at 23–25° and 40–45° are corresponding to 002 and 001 crystallographic planes of turbostratic graphite of BC, respectively. The peaks are slight, which demonstrates that the amorphous phase of BC predominated, and the peaks enhance gradually with the increasing of BC content.

### 3.3. Moisture Content

**Figure 3:**
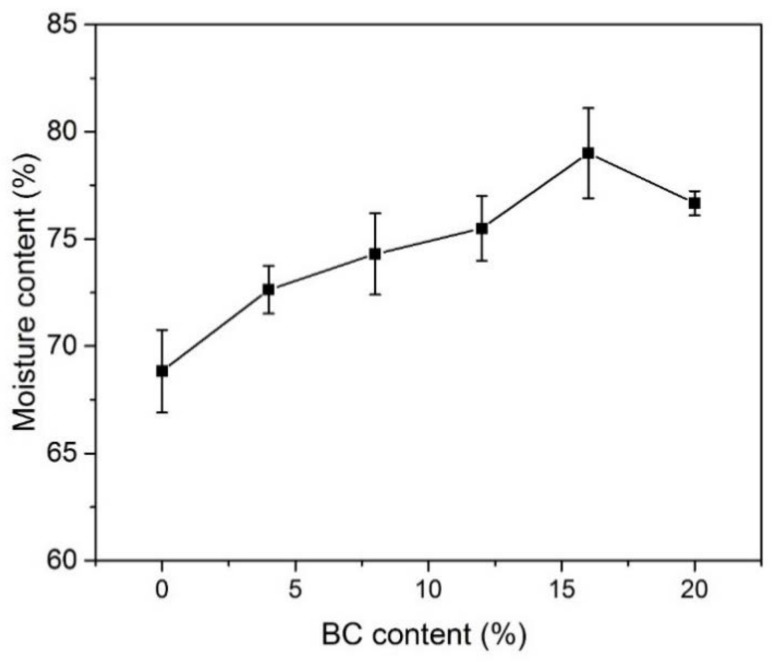
The moisture contents of the PVA-BC-KOH electrolyte membranes with different contents of BC filler (*m_KOH_:m_PVA_* = 2.0:1.5 at 30 °C).

The moisture content of ASPEs is important, because the charge carriers (OH^−^) are taken into the polymer matrix by water molecules and stored in composite membrane via hydrogen bonding or partially in free form. The results of moisture content studies of PVA-BC-KOH (*m_KOH_:m_PVA_* = 2.0:1.5) ASPE membranes are presented in Figure 3. The result indicates that the moisture content reaches a maximum value (79.0 %) with 16 wt % BC content and it decreases below or above this content. In the case of ASPE, there are two methods for the alkaline solution to diffuse into polymer matrix. The solution, in other words, the liquid phase electrolyte can be stored in the inner porous structure of ASPE as shown in the SEM photograph (Figure 1). Meanwhile, PVA shows the ability to absorb water and the amorphous region of PVA matrix can be swelled by the solution to form gel phase electrolyte. The liquid phase electrolyte and the gel phase electrolyte contribute to the conductivity of ASPE together. So, the conductivity represents the similar tendency with the moisture content.

### 3.4. Thermal Analysis

**Figure 4:**
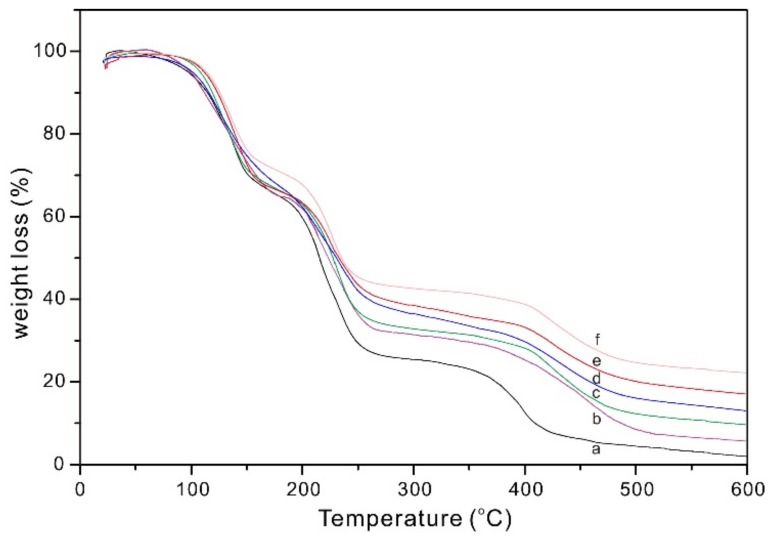
The thermo-gravimetric analysis (TGA) curves of PVA-BC-KOH electrolyte membranes with different contents of BC filler (*m_KOH_*:*m_PVA_* = 2:1.5; a, 0 wt %; b, 4 wt %; c, 8 wt %; d, 12 wt %; e, 16 wt %; f, 20 wt %).

The TGA curves of PVA-BC-KOH ASPEs are shown in Figure 4 with 0, 4, 8, 12, 16 and 20 wt % of BC fillers. Besides the evaporation of water molecules from the polymer matrix at about 100–150 °C, all of the membranes display two weight loss stages at around 160–240 °C and 370–500 °C. The first decomposition temperature of membranes increases from 164 to 172 °C with adding BC of 0–20 wt %, meanwhile, the second one increases from 371 to 423 °C.

The first weight loss was due to the degradation of the polymer membranes; the second one was attributed to the cleavage of the backbone of PVA matrix [25]. On the whole, the degradation of PVA-BC-KOH membranes shifted toward higher temperatures with the content of BC increasing. It means that the flame retardancy of PVA-BC composites is higher compared with pure PVA. This phenomenon can be explained by the presence of BC, firstly, the BC insulated the polymer from heat and thus prevented the degradation. Further, the rough surface of BC in the matrix could block or delay the diffusion of the decomposition products to the surface. Moreover, BC helped to enhance the bond strength in the blended system through co-bridging with the blended polymer to form the net-like structure, and more stable structure required more thermal energy to destroy the bonds in the polymer chains [26]. In addition, the interaction between the BC and the polymer chains, and the retardation effect of BC on the diffusion of free radicals were important factors for improving the thermal stability. It could be concluded that the thermal stability was improved due to the addition of BC.

### 3.5. The Influence of BC Content on Ionic Conductivity

**Figure 5:**
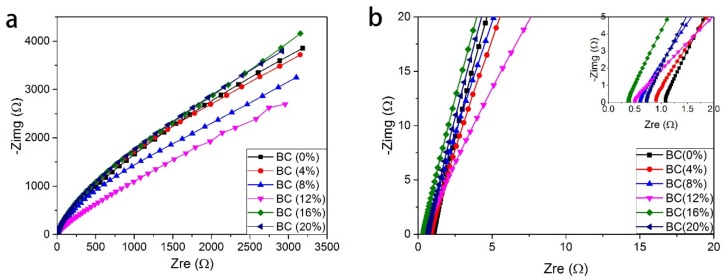
(**a**,**b**) The alternating-current (AC) impedance spectra of the PVA-BC-KOH electrolyte membranes with different contents of BC filler; the inset of b for the high frequency area (*m_KOH_*:*m_PVA_* = 2.0:1.5 at 30 °C).

The typical AC impedance spectra for the PVA-BC-KOH ASPEs with BC fillers of 0–20 wt % are shown in Figure 5a,b. The *R_b_* is taken at the intercept of the Nyquist plot with the real axis. The *R_b_* decreases from 1.09 to 0.38 Ω with the BC fillers content (<16 wt %) increasing. On the contrary, it increases to 0.73 Ω when BC fillers reach 20 wt %. After knowing the *R_b_*, the ionic conductivities of PVA-BC-KOH ASPEs are calculated by Equation (2) based on at least three sample membranes

(2) $$\sigma =\frac{d}{R_{b}\times S}$$

where *d* is the thickness (cm) of the electrolyte sample membrane, *S* is the area of the SS electrode (cm^2^).

**Figure 6:**
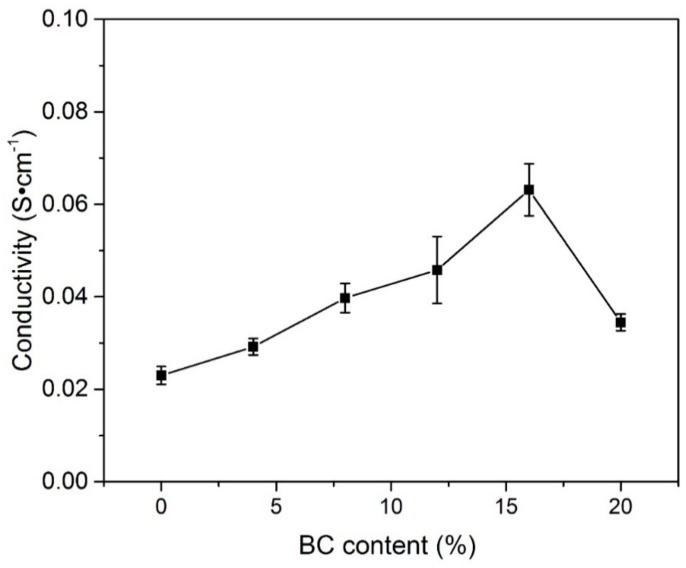
The conductivity of the PVA-BC-KOH electrolyte membranes with different contents of BC filler (*m_KOH_*:*m_PVA_* = 2.0:1.5 at 30 °C).

Figure 6 gives the results of ionic conductivity calculated through Equation (2) for all PVA-BC-KOH ASPEs with various content of BC. When the content of BC is less than 16 wt %, the corresponding ionic conductivity value goes up. It is found from the results that the ionic conductivity heavily relies on the BC content. The maximum average ionic conductivity of 6.63 × 10^−2^ S·cm^−1^ is obtained when the BC filler content is 16 wt %, and the ionic conductivity reduces to 3.45 × 10^−2^ S·cm^−1^ when the BC content achieves 20 wt %.

**Figure 7:**
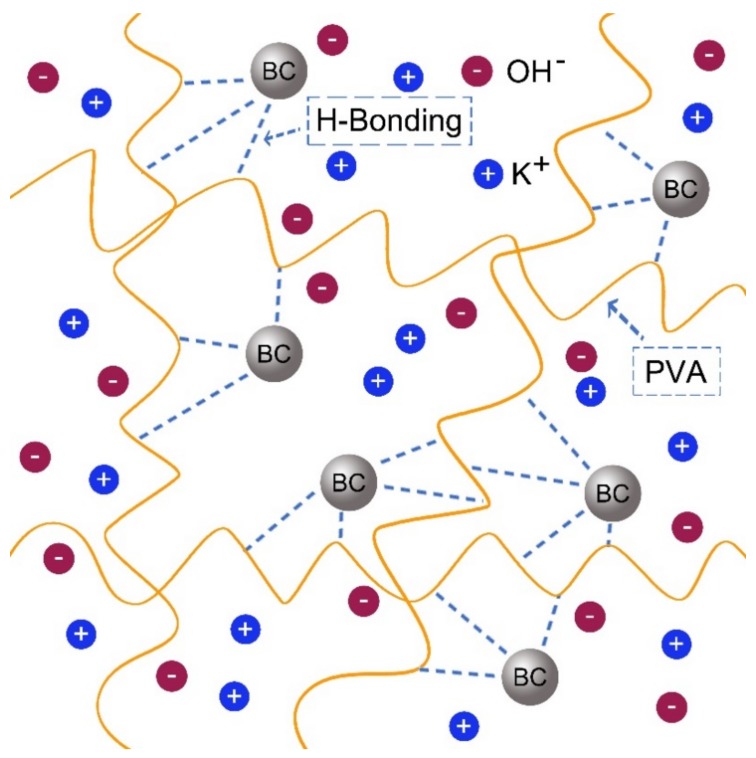
The schematic diagram of PVA-BC-KOH electrolyte membrane structure.

The conductivity of PVA-KOH-BC ASPE originated from two ionic conductive mechanisms: the ions were transferred along the polymer molecular chain through the combination-dissociation process between ions and the polar groups of the polymer [27]; another major conductive factor in the ASPE was the KOH-H_2_O system providing more ion transfer tunnels as a result of the swelling structure and the ions migration increased at higher water content in such system [28]. Such two mechanisms both depended on the crystalline-to-amorphous ratio, which was reduced by the doping of BC. The increase of amorphous region and free-volume could wrap up more water and support local PVA chain segment motion. Therefore, the ions migration increased at higher water content and more ions were transferred in the same time, and finally the conductivity improved. There are many hydroxyl groups existed on the surface of BC and the PVA chain, so the hydrogen bond should be easily formed. And the schematic inner structure model of PVA-KOH-BC ASPE is shown in Figure 7.

The conductivity decreases as the content of BC exceeding 20 wt % since that BC particles precipitation emerges [29,30], and then parts of the ion migration paths are covered. Moreover, the aggregation appeared because of excessive BC filler, therefore, the number of pore deceased within polymer electrolyte, and then the rate of alkali absorption dropped off, leading to the decrease of ionic conductivity directly.

As mentioned in the Introduction section, the ASPE has been studied by some groups, and some good results and mechanisms have been achieved. For example, PVA-TiO_2_-KOH-H_2_O ASPEs with ionic conductivity between 0.102 and 0.171 S·cm^−1^ were prepared by Wu et al. [28]. The function of TiO_2_ filler was to retard or inhibit the recrystallization of PVA polymer and to increase or retain the region of amorphous phase and create more free volume. And the ion transport benefited from such local structural relaxation and segmental motions of the polymer. A more effective promotion was offered by the blending of the ZrO_2_ in PVA ASPE and the highest ionic conductivity was of 0.267 S·cm^−1^ obtained by Yang et al. [29]. It was found that the addition of nano-ZrO_2_ fillers into the PVA polymer matrix could significantly improve the electrochemical properties of ASPE. The researchers thought that the ionic mobility might be increased by defects or free volume at interface between the ZrO_2_ fillers and the PVA polymer matrix. Furthermore, the application of α-Al_2_O_3_ as addition in PVA ASPE was achieved by Mohamad et al. [30], however, the ionic conductivity was only about 10^−7^ S·cm^−1^. The ionic conductivity increased to 10^−4^ S·cm^−1^ with the usage of propylene carbonate, and a local effective pathway for the transport of OH^−^ ion appeared and made the transport faster. Moreover, based on blending tetraethoxysilane (a precursor of SiO_2_ filler) and crosslinking through glutaraldehyde, the PVA-SiO_2_ nanocomposite polymer membranes with remarkable electronic performance was achieved by Yang et al. [31]. The ionic conductivity was 0.035 S·cm^−1^ with 10 wt % SiO_2_ at ambient temperature. The SiO_2_ nanoparticles acted as a solid plasticizer capable of enhancing the chemical and thermal properties, and improve the dimensional stability. And the many hydroxide groups on the SiO_2_ filler surfaces greatly enhanced the KOH retaining ability.

### 3.6. The Influence of Content of KOH on Ionic Conductivity

**Figure 8:**
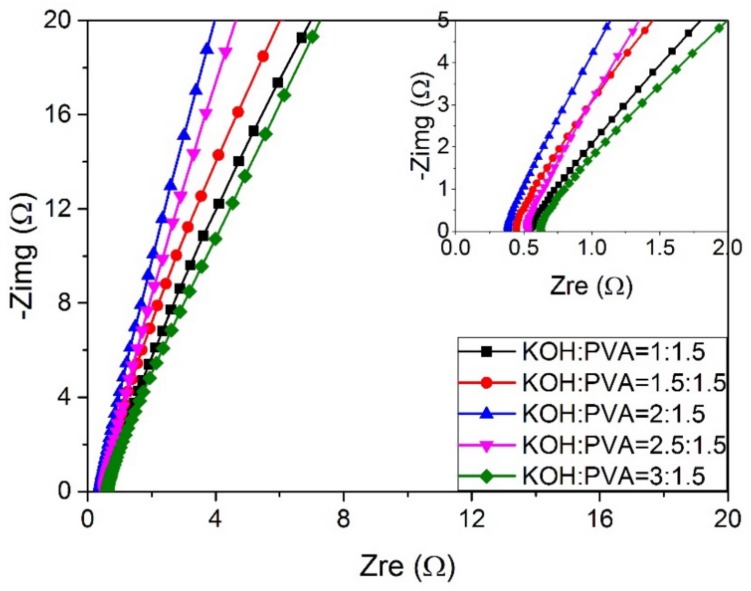
The AC impedance spectra of the PVA-BC-KOH electrolyte membranes with different contents of KOH; the inset for the high frequency area (BC 16 wt % at 30 °C).

**Figure 9:**
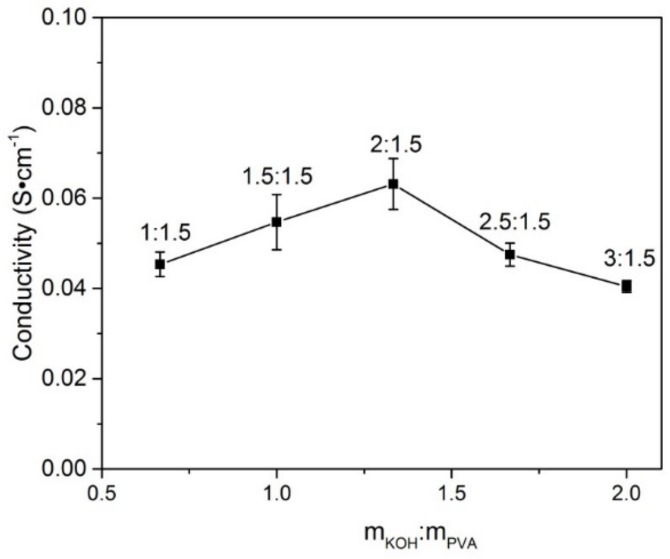
The conductivity of the PVA-BC-KOH electrolyte membranes with different contents of KOH (BC 16 wt % at 30 °C).

As shown in Figure 8, the conductivity of PVA-16 wt % BC-KOH ASPEs varies with different contents of KOH. Figure 9 gives the results of ionic conductivity at 30 °C calculated from Equation (2). As the ratio of KOH to PVA increases, the *R_b_* values decrease, and then the ionic conductivities rise. The electrolyte membrane has the lowest *R_b_* value of 0.42 Ω and the best ionic conductivity of 6.65 × 10^−2^ S·cm^−1^ with the *m_KOH_*:*m_PVA_* of 2.0:1.5. Nevertheless, when the ratio of *m_KOH_*:*m_PVA_* exceeds 2.0:1.5, the ionic conductivity begins to decrease. That is, the higher KOH content does not make a further contribution to the conductivity.

The increase of conductivity could be attributed to the increase of ion charge carrier. The charge carriers (OH^−^) were taken into the polymer by water molecules and stored in composite membrane via hydrogen bonding between hydroxy groups of PVA and KOH [23]. The concentration of conductive ions in the membrane rose with the content of KOH increasing, and hence the conductivity increased [28]. High concentrations of KOH could lead to the appearance of crystalline phase in the polymer membrane system and make the polymer chains inflexible, which greatly reduced the migration ability of OH^−^ in the membrane [32].

### 3.7. Influence of Temperature on the Ionic Conductivity

**Figure 10:**
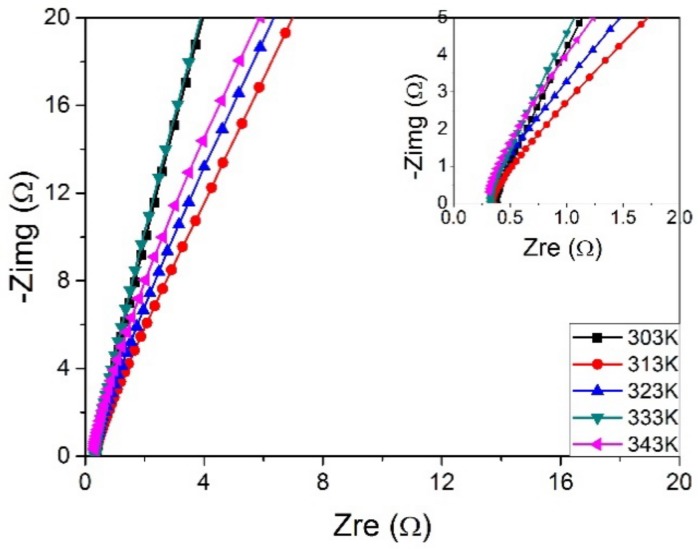
The AC impedance spectra of the PVA-BC-KOH electrolyte membranes with different temperatures; the inset for the high frequency area (BC 16 wt %, *m_KOH_*:*m_PVA_* = 2.0:1.5).

The ionic conductivity continuously increases with the rise of temperature. The ionic conductivities of PVA-16 wt % BC-KOH electrolyte membrane at different temperatures are calculated based on Figure 10 with *m_KOH_*:*m_PVA_* = 2.0:1.5. The electrolyte membrane possesses ionic conductivity of 0.069–0.084 S·cm^−1^ in the temperature range 303–343 K.

It was observed that the ionic conductivity of polymer electrolyte increases as a function of temperature. Firstly, at higher temperature, the thermal motion of polymer chain segments would be improved, which increased ions transport speed and the numbers of transport tunnels [28]; moreover, as the temperature increases, the more structural relaxation of the polymer chains would expand free-volume, which contributed to an improvement of ion migration ability. Besides, the viscosity and diffuse resistance also had an important effect on ionic transport and, obviously, they decline with the increasing of temperature, which improved electric conductivity to some degree [33].

**Figure 11:**
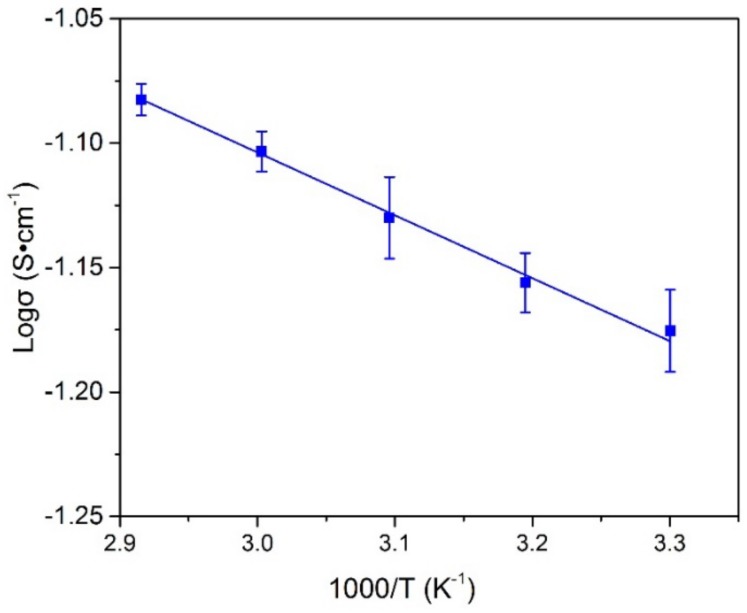
Arrhenius Plot of PVA-BC-KOH electrolyte membranes with different temperatures.

The logσ displays a linear relationship with the 1000/T as shown in Figure 11. Therefore, the temperature dependence of ionic conductivity follows the Arrhenius law (Equation (3)):

(3) $$\sigma =A\;\exp(-\frac{E_{a}}{R\times T})$$

where *A* is the pre-exponential, *E_a_* is the activation energy (kJ·mol^−1^), *R* is the universal gas constant (J·mol^−1^·K^−1^), and *T* is the absolute temperature (K).

The activation energy (*E_a_*) value of PVA-BC-KOH ASPE is about 4.35 kJ·mol^−1^ calculated from the Equation (3), which is lower than the pure PVA-KOH ASPE as 4.78 kJ·mol^−1^ [18]. This can be due to the fact that activation energy is related to its ionic mobility. The crystallization of PVA-BC-KOH ASPE membrane is hindered by doping with BC filler, generating more amorphous phase, which can decrease the energy barrier of ionic transport and facilitate the fast ion migration.

### 3.8. The Electrochemical Stability Windows

**Figure 12:**
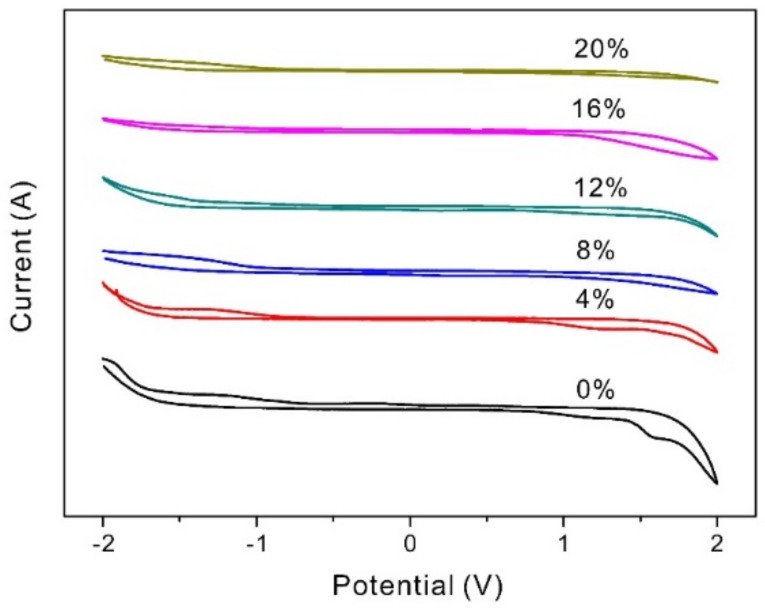
The cyclic voltammetry curves of the PVA-BC-KOH electrolyte membranes with different contents of BC filler (*m_KOH_*:*m_PVA_* = 2.0:1.5 at 30 °C).

The electrochemical stability window is considered one of the most important factors in practical applications of ASPE. It is defined as a region of potential without the flowing of the Faraday induced current through the electrolyte [34], and it is limited in its cathodic and anodic parts, where the reduction and oxidation of the polymer electrolytes and the OH^−^ ions take place. The cyclic voltammogram of PVA-BC-KOH ASPEs with different contents of BC are shown in Figure 12 in the voltage range of −2–2 V. The electrochemical stability window is 1.54 V without BC and it increases to about 1.7 V after the addition of 8 wt % BC. Then it is found that the addition of BC has little effect on the electrochemistry stability, and the electrochemical stability windows are in the range of 1.67–1.7 V, which are permissible for practical applications. The better electrochemistry stability is obtained after the adding of BC because the interface area between PVA-BC-KOH ASPE and the blocking electrolytes contained much more KOH electrolyte [31].

### 3.9. The Applications in Ni-MH Battery

**Figure 13:**
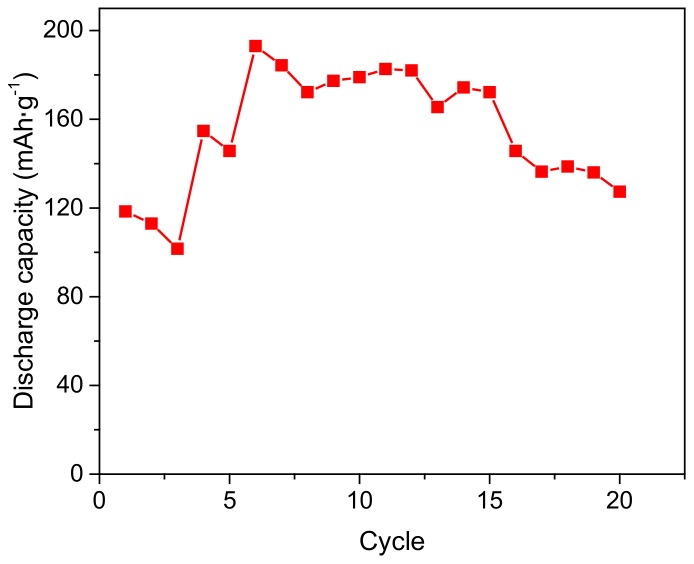
The discharge capacity of the assembled Ni-MH batteries.

Figure 13 shows the discharge capacity as a function of the cycle number of the Ni-MH battery. The comparatively low capacity value at the beginning of the discharge is related to the electrode-electrolyte contact [35], which is not always favorable during the 1–5th cycles. It is observed that the discharge capacity can reach the maximum of 193 mAh·g^−1^, and the battery has an average discharge capacity of 180 mAh·g^−1^ at 6–12th cycles. After 12 cycles, the discharge capacity sharply declines, which can be attributed to the reaction between the battery negative electrode and air, resulting in more corrosion inside of the negative electrode. The Ni-MH battery in this experiment was simply assembled without airtight packing, which decreased the capacity and cycle life. However, the feasibility of the PVA-BC-KOH polymer electrolyte membrane for Ni-MH battery still could be proved.

## 4. Conclusions

The PVA-KOH based ASPE with the natural BC filler was prepared by solution casting technique. The highest conductivity value of 6.63 × 10^−2^ S·cm^−1^ was obtained with the *m_KOH_*:*m_PVA_* of 2:1.5 and 16 wt % content of BC. The addition of BC could hinder the crystallization of PVA, and the ions migration increased accompanying the increase of amorphous region and free-volume, which resulted in the increase in conductivity. The high water content could be determined by the measurement of moisture content and the porous structure observed by SEM, which contained much KOH electrolyte and was beneficial to conductivity. The electrochemical stability window also expanded after the addition of BC to 1.7 V, and then changed a little with the increase of BC content. A simple Ni-MH battery containing PVA-BC-KOH electrolyte membrane was assembled with the maximum discharge capacity of 193 mAh·g^−1^ and the feasibility of the ASPE could be proved.

## Supplementary Materials

The following are available online at http://www.mdpi.com/1996-1944/11/5/679/s1, Table S1: The dosages of PVA, BC and KOH of PVA-BC-KOH ASPE membranes.

## References

[1] Yang Y., Liu X., Dai Z., Yuan F., Bando Y., Golberg D., Wang X. (2017). In situ electrochemistry of rechargeable battery materials: Status report and perspectives. Adv. Mater..

[2] Sebastián D., Baglio V. (2017). Advanced materials in polymer electrolyte fuel cells. Materials.

[3] Ryu H., Lee J.H., Kim T.Y., Han U.K., Lee J.H. (2017). High-performance triboelectric nanogenerators based on solid polymer electrolytes with asymmetric pairing of ions. Adv. Energy Mater..

[4] Novitski D.M., Kosakian A., Weissbach T., Secanell M., Holdcroft S. (2016). Electrochemical reduction of dissolved oxygen in alkaline solid polymer electrolyte films. J. Am. Chem. Soc..

[5] Yu R., Bao J.J., Chen T.T., Zou B.K., Wen Z.Y., Guo X.X., Chen C.H. (2017). Solid polymer electrolyte based on thermoplastic polyurethane and its application in all-solid-state lithium ion batteries. Solid State Ion..

[6] Yuan A., Zhao J. (2006). Composite alkaline polymer electrolytes and its application to nickel–metal hydride batteries. Electrochim. Acta.

[7] Iwakura C., Nohara S., Furukawa N., Inoue H. (2002). The possible use of polymer gel electrolytes in nickel/metal hydride battery. Solid State Ion..

[8] Geng M., Northwood D.O. (2003). Development of advanced rechargeable Ni/MH and Ni/Zn batteries. Int. J. Hydrog. Energy.

[9] Albert A., Lochner T., Schmidt T.J., Gubler L. (2016). Stability and degradation mechanisms of radiation-grafted polymer electrolyte membranes for water electrolysis. ACS Appl. Mater. Interfaces.

[10] Fattah N.F.A., Ng H.M., Mahipal Y.K., Numan A., Ramesh S., Ramesh K. (2016). An approach to solid-state electrical double layer capacitors fabricated with graphene oxide-doped, ionic liquid-based solid copolymer electrolytes. Materials.

[11] Hassan M.F., Arof A.K. (2005). Ionic conductivity in PEO-KOH polymer electrolytes and electrochemical cell performance. Phys. Status Solidi.

[12] Chandrasekaran R., Mangani I.R., Vasanthi R., Selladurai S. (2001). Ionic conductivity and battery characteristic studies on PEO + NaClO_3_, polymer electrolyte. Ionics.

[13] Yang C.C., Lin S.J., Hsu S.T. (2003). Synthesis and characterization of alkaline polyvinyl alcohol and poly (epichlorohydrin) blend polymer electrolytes and performance in electrochemical cells. J. Power Sour..

[14] Virya A., Lian K. (2017). Polyacrylamide-lithium chloride polymer electrolyte and its applications in electrochemical capacitors. Electrochem. Commun..

[15] Liao G.M., Yang C.C., Hu C.C. (2018). Optimal loading of quaternized chitosan nanofillers in functionalized polyvinyl alcohol polymer membrane for effective hydroxide ion conduction and suppressed alcohol transport. Polymer.

[16] Lewandowski A., Skorupska K., Malinska J. (2000). Novel poly (vinyl alcohol)-KOH-H_2_O alkaline polymer electrolyte. Solid State Ion..

[17] Li J., Lian K. (2016). A comparative study of tetraethylammonium hydroxide polymer electrolytes for solid electrochemical capacitors. Polymer.

[18] Yang C.C. (2004). Chemical composition and XRD analyses for alkaline composite PVA polymer electrolyte. Mater. Lett..

[19] Ngai K.S., Ramesh S., Ramesh K., Juan J.C. (2016). A review of polymer electrolytes: Fundamental, approaches and applications. Ionics.

[20] Pan W.H., Lue S.J., Chang C.M., Liu Y.L. (2011). Alkali doped polyvinyl alcohol/multi-walled carbon nano-tube electrolyte for direct methanol alkaline fuel cell. J. Membr. Sci..

[21] Hema M., Tamilselvi P., Pandaram P. (2017). Conductivity enhancement in SiO_2_ doped PVA: PVDF nanocomposite polymer electrolyte by gamma ray irradiation. Nucl. Instrum. Methods Phys. Res. Sect. B.

[22] More S., Dhokne R., Moharil S. (2017). Structural properties and temperature dependence dielectric properties of PVA-Al_2_O_3_, composite thin films. Polym. Bull..

[23] Zhang W., Liu X., Wang D., Jin Y. (2017). Effects of bamboo charcoal on fouling and microbial diversity in a flat-sheet ceramic membrane bioreactor. Bioresour. Technol..

[24] Ma C.A., Xu C., Shi M., Song G., Lang X. (2013). The high performance of tungsten carbides/porous bamboo charcoals supported Pt catalysts for methanol electrooxidation. J. Power Sour..

[25] Yang C.C. (2012). Alkaline direct methanol fuel cell based on a novel anion-exchange composite polymer membrane. J. Appl. Electrochem..

[26] Xiao W., Li X.H., Wang Z.X., Guo H.J., Wang J.X., Huang S.L., Gan L. (2012). Physicochemical properties of a novel composite polymer electrolyte doped with vinyltrimethoxylsilane-modified nano-La_2_O_3_. J. Rare Earth.

[27] Zhang J., Han H., Wu S., Xu S., Yang Y., Zhou C., Zhao X. (2007). Conductive carbon nanoparticles hybrid PEO/P(VDF-HFP)/SiO_2_, nanocomposite polymer electrolyte type dye sensitized solar cells. Solid State Ionics.

[28] Wu Q., Zhang J., Sang S. (2008). Preparation of alkaline solid polymer electrolyte based on PVA-TiO_2_-KOH-H_2_O and its performance in Zn-Ni battery. J. Phys. Chem. Solids.

[29] Yang C.C. (2006). Study of alkaline nanocomposite polymer electrolytes based on PVA–ZrO2–KOH. Mater. Sci. Eng. B.

[30] Mohamad A.A., Arof A.K. (2007). Plasticized alkaline solid polymer electrolyte system. Mater. Lett..

[31] Yang C.C., Li Y.J., Liou T.H. (2011). Preparation of novel poly(vinyl alcohol)/SiO_2_ nanocomposite membranes by a sol–gel process and their application on alkaline DMFCs. Desalination.

[32] Li B., Lu X., Yuan J., Zhu Y., Li L. (2015). Alkaline poly(vinyl alcohol)/poly(acrylic acid) polymer electrolyte membrane for Ni-MH battery application. Ionics.

[33] Popa S., Lliescu S., Llia G., Plesu N., Popa A., Visa A., Macarie L. (2017). Solid polymer electrolytes based on phosphorus containing polymers for lithium polymer batteries. Eur. Polym. J..

[34] Fan L.D., Chen J., Qin G., Wang L.B., Hu X.Y., Shen Z.S. (2017). Preparation of PVA-KOH-Halloysite nanotube alkaline solid polymer electrolyte and its application in Ni-MH battery. Int. J. Electrochem. Sci..

[35] Mohamad A.A., Mohamed N.S., Yahya M.Z.A., Othman R., Ramesh S., Alias Y., Arof A.K. (2003). Ionic conductivity studies of poly(vinyl alcohol) alkaline solid polymer electrolyte and its use in nickel-zinc cells. Solid State Ion..

