# Supplementary material for: Preparation and Characterization of PVA Alkaline Solid Polymer Electrolyte with Addition of Bamboo Charcoal
Source: Materials (Basel). 2018 Apr 26;11(5):679. doi: 10.3390/ma11050679 (PMC5978056; doi:10.3390/ma11050679)
Supplement: Supplementary file 1 [file materials-11-00679-s001.pdf]

Supporting Information

Preparation and Characterization of PVA Alkaline Solid Polymer Electrolyte with Addition of Bamboo Charcoal

Lidan Fan [1], Mengyue Wang [2], Zhen Zhang [2], Gang Qin [2,*], Xiaoyi Hu [2], Qiang Chen [2,*]

[1] School of Civil Engineering, Henan Polytechnic University, Jiaozuo 454001, China; lidanfan@hpu.edu.cn

[2] School of Materials Science and Engineering, Henan Polytechnic University, Jiaozuo 454001, China, wangmengyue1111@sina.com (M.W.); zhangergou1014@sina.com (Z.Z.); yizhixinhai_hu@163.com (X.H.)

* Correspondence: qingang@hpu.edu.cn; Tel.: +8615978769169 (G.Q.); chenqiang@hpu.edu.cn; Tel.: +8619939106934 (Q.C.)

Table S1. The dosages of PVA, BC and KOH of PVA-BC-KOH ASPE membranes

PVA/g	BC/g	KOH/g
1.5	0	2
1.5	0.063	2
1.5	0.13	2
1.5	0.205	2
1.5	0.286	2
1.5	0.375	2
1.5	0.286	1
1.5	0.286	1.5
1.5	0.286	2.5
1.5	0.286	3
